# Short-, long-, and very long-term results of secondary anterior sphincteroplasty in 20 patients with obstetric injury

**DOI:** 10.1007/s00384-021-04026-1

**Published:** 2021-09-16

**Authors:** Helene Marie Haug, Erik Carlsen, Hans-Olaf Johannessen, Egil Johnson

**Affiliations:** 1 Department of Pediatric and Gastrointestinal Surgery, Oslo University Hospital, Ullevål, P. O. Box 4956, 0424 Nydalen, Oslo Norway; 2 Institute of Clinical Medicine, University of Oslo, Oslo, Norway

**Keywords:** Obstetric injury, Anal incontinence, Secondary sphincteroplasty, Long-term outcome

## Abstract

**Purpose:**

More long-term follow-up studies beyond 10 years after secondary sphincteroplasty for obstetric damage are warranted. This prospective study aimed to compare reported data on incontinence and satisfaction in a cohort of such patients examined at short-, long-, and very long-term follow-up.

**Methods:**

Twenty out of 33 obstetric patients (61%) operated with secondary anterior overlapping sphincteroplasty during February 1996 to April 2004 were evaluated preoperatively and at short-, long-, and very long-term follow-up. Anal incontinence was scored by a combination of Wexner’s and St. Mark’s incontinence scores. The patients also reported degree of treatment satisfaction.

**Results:**

Twenty patients were examined preoperatively and after a median (range) of 5 (2–62), 102 (64–162), and 220 (183–278) months. Corresponding incontinence scores were 11.5 (5–18), 5.5 (1–17) (*p* < 0.01), 10.0 (0–18) (*p* > 0.05), and 12.0. (1–18) (*p* > 0.05). With increasing follow-up times, patients reporting a better outcome were 75%, 65%, and 45%. At very long-term follow-up patients, reports were more dismal than expected in those also reporting improved incontinence cores. Incontinence scores did not improve in patients with neuropathy (*n* = 5) or patients (*n* = 5) with more than 10 years of symptoms.

**Conclusion:**

Initial improvement of anal incontinence attenuated with time, in particular from short- to long-term follow-up. Patients with neuropathy experienced no improvement of incontinence. Beyond stoma formation, in compliant patients, one should consider other treatment options like sacral nerve stimulation and neosphincter formation.

## Introduction

The reported incidence of overt obstetric anal sphincter injuries in the Nordic countries varies from 0.6 to 4.2% [1]. However, there is evidence of sphincter defects after vaginal delivery in primiparous women evaluated by 3D ultrasound in around 11% and 8,5% in subsequent deliveries [2]. The short-term success rate of secondary repair of sphincter defects after obstetric trauma lies between 64 and 80% [3–5]. Several studies have shown a deterioration of improved incontinence over time. A review of 16 studies with a median observation time of 58–129 months reported good or excellent outcomes in about 50%. Predictors of long-term failure included advanced age, duration of symptoms, and pudendal neuropathy [6].

In 2010 we reported short- and long-term outcomes (102 months) in 33 obstetric women following treatment of anal incontinence with secondary anterior sphincteroplasty [7]. This study aimed to report results after a very long-term follow-up of these patients.

## Material and methods

Twenty of the thirty-three patients (61%) reported in 2010 were eligible for very long-term follow-up in March 2019. Three patients were deceased. Ten patients were excluded because of not responding in seven and needing a stoma in three.

Preoperative ambulatory workup included clinical evaluation (*n* = 20), recording of incontinence scores (*n* = 20), manometry (*n* = 18), ultrasonography (*n* = 17), pudendal nerve terminal motor latency (PNTML) (*n* = 9), and needle electromyography (EMG) (*n* = 9). A PNTML ≥ 2.3 ms. and needle EMG showing polyphasic potentials and signs of denervation and reinnervation were compatible with neuropathy. At short-term follow-up, patients were scored for incontinence and patient satisfaction (excellent, improved, unaltered, worse) in collaboration with the physician at the ambulatory unit. At long term and very long term, patients were evaluated by phone interview and by completing a questionnaire returned by mail. Incontinence was evaluated using a combination of Wexner’s and St. Mark’s scores. Parameters reported at all follow-ups were leakage of air, leakage of liquid stool, solid stool, and lifestyle alteration expressed as seldom (1 point), monthly (2 points), weekly (3 points), and daily (4 points). Use of pad expressed as yes (2 points) and no (0 points); complete incontinence giving a maximum score of 18.

The median number of vaginal births was 2 (range 1–4). Eleven had an overt rupture of the anal sphincters. Of these, three had delivery by vacuum extraction and breech birth in one. Three had episiotomy performed during delivery. Judged by peroperative findings and anal ultrasonography, all patients had third or fourth-degree sphincter tear: grade 3a (*n* = 1), 3b (*n* = 11), 3c (*n* = 6), or 4 (*n* = 2).

The patients were operated with an adapting and overlapping sphincteroplasty of the external sphincter, including puborectal sling adaptation, using slowly resorbable monofil polydioxanone 2–0 sutures, [7]. The skin was adapted with an intracutaneous resorbable polyfilament suture over a subcutaneous drain. An experienced consultant always participated in the operations. Three patients (15%) had more than one operation. One had repeat anal reefing and plasty of the puborectal sling after 46 months. The second had anal reconstruction after 78 months, followed by separate suturing of the internal and external sphincter after 210 months at another hospital. The third patient had a sphincteroplasty with separate suturing at another hospital after approximately 144 months.

Comparison of incontinence scores was made with non-parametric repeated measures using ANOVA (Friedman multiple comparisons test). We used Graphpad Instat version 3.0 for Windows (San Diego, USA), and *p* values < 0.05 were defined as significant.

## Results

At initial sphincteroplasty median (range) age and duration of symptoms for the 20 patients were 36 years (29–59) and 3 (1–20) years. They were examined at short-, long-, and very long-term follow-up after median (range) 5 (2–62), 102 (64–162), and 220 (183–278) months. Median (range) incontinence scores preoperatively, after short-, long-, and very long-term evaluation, were 11.5 (5–18), 5.5 (1–17) (*p* < 0.01), 10.0 (0–18) (*p* > 0.05), and 12.0. (1–18) (*p* > 0.05).

Incontinence score was matched with patient satisfaction. At short-term follow-up, the percentage of patients reporting normalized score/excellent results were 10/10, reduced score/improved result 70/65, unaltered/unaltered 5/20, and increased score/worse result 15/5. At long term, the numbers were 15/15, 50/50, 10/30, and 25/5, and at very long term, 5/10, 60/35, 10/30, and 25/25. Interestingly, 25% of the patients reported a deteriorated treatment result at a very long-time evaluation despite a persistent incontinence score. During the 18 years of observation time, the fraction of patients reporting excellent and improved results declined from 75 to 45%.

Eleven (55%), 8 (40%), 11 (55%), and 10 (50%) patients wore a pad before operation and after the respective times of follow-up. Corresponding figures for patients using constipating medicines were 5%, 5%, 15%, and 10%. After long- and very long-term follow-up, 11 (55%) and 10 (50%) of the patients could defer defecation for 15 min or more.

**Fig. 1:**
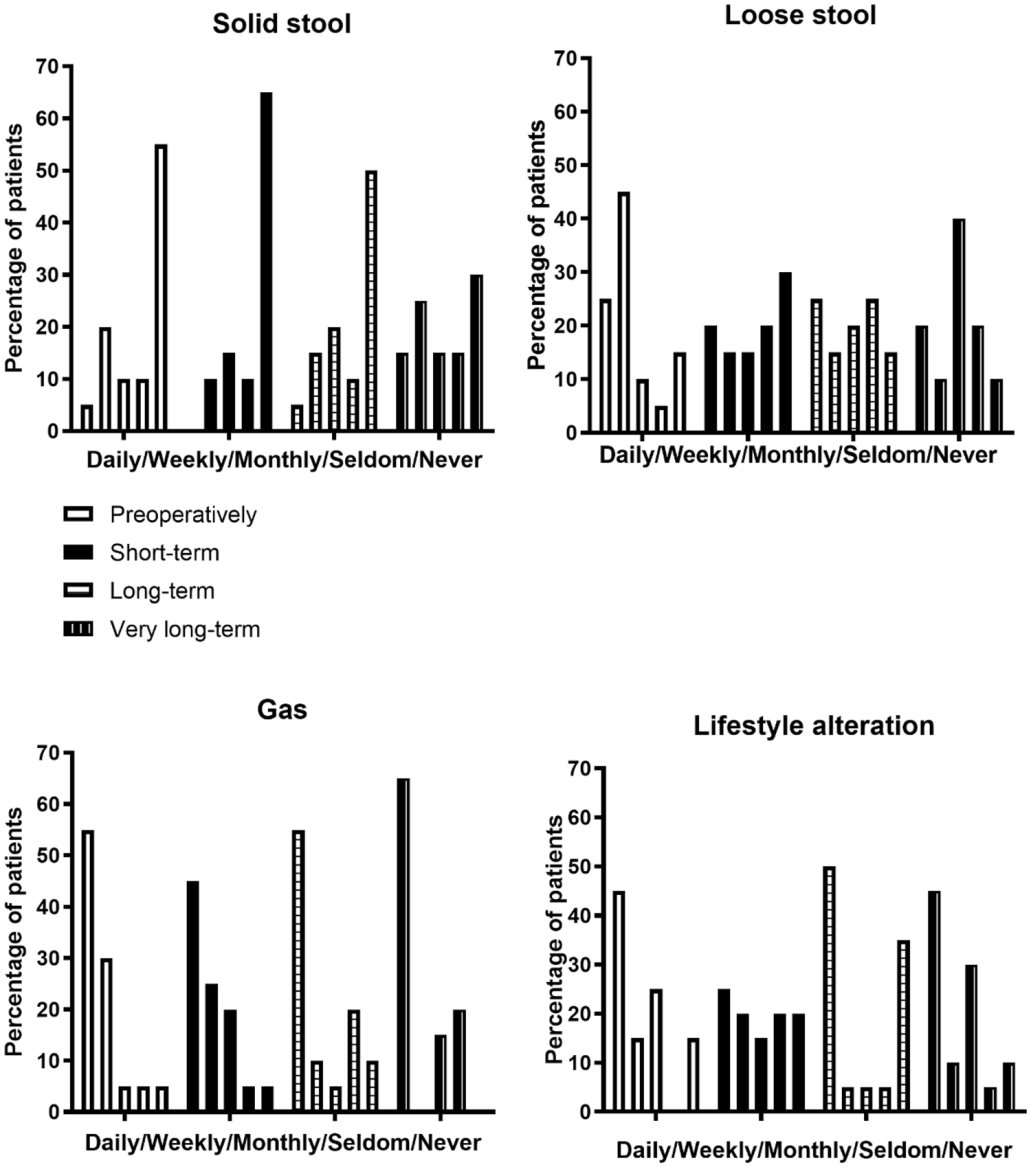
Percentage of the 20 patients with and without anal leakage and lifestyle alteration preoperatively and after short-, long- and very long-term follow-up

Results concerning the degree of incontinence and lifestyle alteration preoperatively and at follow-up are shown in Fig. 1. There was no longer any improvement of continence concerning solid and liquid stool after very long-time follow-up compared with before operation.

The patients were analyzed for incontinence scores related to the degree of sphincter tear without revealing striking differences in incontinence. Grade 3a (*n* = 1) had incontinence score of median (range) 17 preoperatively, 17 at short term, 13 at long term, and 15 at very long term. Grade 3b (*n* = 11), 11 (6–17), 8 (1–15), 12 (1–16), 12 (1–17). 3c (*n* = 6), 11 (5–16), 5,5 (1–13), 7 (2–14), 10,5 (2–18). Grade 4 (*n* = 2): 13,5 (12–15), 8 (2–14), 11,5 (6–17), 11 (8–14).

Five patients without neuropathy, who had very long symptom duration of median 15 years (range 10–20), proved to have little effect of operation. Respective scores were 14 (11–19), 13 (7–16), 15 (6–18), and 17 (12–20).

In patients without known neuropathy (*n* = 15), the preoperative and post-operative incontinence scores with increasing follow-up times were 11 (5–16), 5 (1–13) (*p* < 0.05), 6 (0–15) (*p* > 0.05), and 9 (1–18) (*p* > 0.05), respectively. In patients (*n* = 5) with neuropathy, corresponding scores were 16 (13–18), 13 (8–17) (*p* > 0.05), 15 (14–18) (*p* > 0.05), and 14 (12–17) (*p* > 0.05).

## Discussion

After very long-term follow-up after secondary sphincter repair for obstetric damage, the main result was that improved continence deteriorated more strongly from short- to long-time (4.5 points) versus long- to very long-time (2 points) follow-up.

However, in the 15 patients without neuropathy, although not significant, there probably was a treatment effect with improved continence after both 8.5 years (5 points) and 18 years (2 points) of follow-up. The pattern of reduced continence as a function of observation times, mostly from 10 to 120 months, but also after 18 years, was consistent with other reports [8]. In line with other studies [6], the precise control of continence for flatus was not improved by sphincteroplasty.

The five patients with neuropathy based on delayed PNTML and needle EMG had no definitive improvement of continence. Still, the scores remained stable at long and very long-term follow-up, supporting that sphincteroplasty per se consolidated the sphincter.

The discrepancy at very long-term evaluation reported between reduced incontinence score in 65% and improved treatment results in only 45% of the patients could partly be owing to a negative psychological effect because of many years with persisting incontinence. However, at the ultimate evaluation, only half of the patients wore a pad, comparable with both before operation and after long time evaluation. This corresponds with findings that the major decline of incontinence took place from short- to long-time follow-up (Fig. 1). However, without operation, these patients undoubtedly would have been even worse off regarding the degree of incontinence.

We found that degree of sphincter tear did not have any crucial influence on the treatment results in this cohort of 20 patients. On the contrary, five patients with long symptom duration without neuropathy had no symptomatic benefit of sphincteroplasty. This is consistent with other reports [9].

Reasons for early failure of a sphincteroplasty can be lack of a tension-free repair and complications from hematoma, wound infection, and fecal impaction. Crucial factors for long-term failure are less evident but could be denervation of the external sphincter muscle during dissection [10] and time-dependent muscular fibrosis and atrophy.

Further treatment options, particularly for older women with progressing intolerable long-term incontinence, could be placement of a stoma. Alternatively, in younger and compliant patients’ sacral nerve, stimulation or formation of a neosphincter by dynamic graciloplasty or artificial bowel sphincter is potential but also complex treatment options with inherent complications and low success rate [2, 11].

## Conclusion

In conclusion, even after 18 years, 20 out of 33 patients (61%) reported data after secondary sphincteroplasty. The incontinence and lifestyle alteration results most strongly declined from short- to long-term follow-up. The degree of incontinence reached the preoperative level at the hitherto almost unparalleled very long-term follow-up of 18 years. Although not statistically significant, patients without neuropathy still had an improved result at ultimate follow-up. Thus, secondary sphincteroplasty for obstetric damage is worthwhile, but the effect deteriorates, and one should consider additional treatment options in selected patients.

## Data Availability

Data available on request.

## Code availability

We used the statistical program Graphpad Instat version 3.0 for Windows (San Diego, USA).
